# Genecentric: a package to uncover graph-theoretic structure in high-throughput epistasis data

**DOI:** 10.1186/1471-2105-14-23

**Published:** 2013-01-18

**Authors:** Andrew Gallant, Mark DM Leiserson, Maxim Kachalov, Lenore J Cowen, Benjamin J Hescott

**Affiliations:** 1 Department of Computer Science, Tufts University, Medford, MA 02155, USA; 2 Department of Computer Science, Brown University, Providence, RI 02912, USA

## Abstract

**Background:**

New technology has resulted in high-throughput screens for pairwise genetic interactions in yeast and other model organisms. For each pair in a collection of non-essential genes, an epistasis score is obtained, representing how much sicker (or healthier) the double-knockout organism will be compared to what would be expected from the sickness of the component single knockouts. Recent algorithmic work has identified graph-theoretic patterns in this data that can indicate functional modules, and even sets of genes that may occur in compensatory pathways, such as a BPM-type schema first introduced by Kelley and Ideker. However, to date, any algorithms for finding such patterns in the data were implemented internally, with no software being made publically available.

**Results:**

Genecentric is a new package that implements a parallelized version of the Leiserson et al. algorithm (J Comput Biol 18:1399-1409, 2011) for generating generalized BPMs from high-throughput genetic interaction data. Given a matrix of weighted epistasis values for a set of double knock-outs, Genecentric returns a list of generalized BPMs that may represent compensatory pathways. Genecentric also has an extension, GenecentricGO, to query FuncAssociate (Bioinformatics 25:3043-3044, 2009) to retrieve GO enrichment statistics on generated BPMs. Python is the only dependency, and our web site provides working examples and documentation.

**Conclusion:**

We find that Genecentric can be used to find coherent functional and perhaps compensatory gene sets from high throughput genetic interaction data. Genecentric is made freely available for download under the GPLv2 from http://bcb.cs.tufts.edu/genecentric.

## Background

When two non-essential genes are simultaneously deleted, sometimes a surprising phenotype emerges compared to the phenotype of the deletion mutants of the single genes. When studying the yeast genome, often this can be quantified in terms of the growth rate of the double deletion mutant, compared to the growth rate of its component single deletion mutants, termed epistasis [1]. Recent SGA [2], dSLAM [3] and E-MAP [4] technology produces high throughput weighted epistasis values for large collections of double knockouts.

A variety of algorithmic methods have been proposed to infer functionally meaningful relationships between genes based on the structure of their epsistatic genetic interactions [5-10]. In particular, we consider the generalized “Between Pathway Model” (or BPM) as studied by Leiserson *et al*[5]. As discussed in [5], this involves finding pairs of sets of genes, where the sum of the epistasis values between genes in different sets, minus the sum of the epistasis values between genes in the same set, is as negative as possible. This is a generalization of an unweighted BPM model studied by Ma et al. [11] and Brady et al. [12], which was a simplification of the original BPM model introduced by Kelley and Ideker [13]. Other versions of BPMs, in different settings or criteria, have been studied [9,14,15].

Leiserson et al. [5] presented a randomized algorithm based on maximal graph cuts to generate these putative generalized BPMs from weighted epistasis data. They showed that the BPMs produced by their method were biologically enriched when their method was run on several different yeast E-MAP and SGA data sets [8,16]. We now present a full implementation, Genecentric, of this algorithm that is fast, easy to use, well documented and open source. In addition, Genecentric has an extension that performs GO enrichment analysis using FuncAssociate’s web service API [17].

## Implementation

Genecentric implements a randomized algorithm to generate putative generalized BPMs as described in Leiserson *et al*[5].

### Algorithm

Genecentric takes as input a file with genetic interaction data and outputs a list of pairs of sets of genes corresponding to the putative generalized BPMs. The genetic interaction data must be in a tab-delimited format where each row corresponds to a gene pairing and that pairing’s interaction score. Specifically, the first column contains the first gene identifier, the second column contains the second gene identifier, and the third column contains the interaction score.

**Figure 1:**
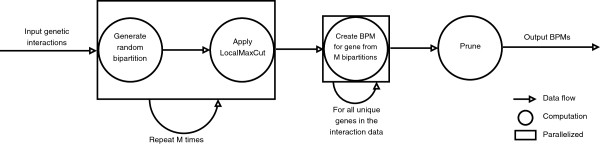
**Graphical representation of the algorithm implemented by Genecentric.** Edges represent data flow, circles represent computation and rectangles envelop independent computations that can be parallelized in the presences of multiple cores.

We’ll now brielfy recap the algorithm described in Leiserson *et al*[5]. (Figure 1 provides a graphical representation of the algorithm.) First, the input genetic interaction data is used to create *M* random bipartitions, where the vertices correspond to genes and an edge between genes corresponds to its interaction score. Second, each random bipartition is transformed into a LocalMaxCut by iteratively modifying it until every vertex in the graph satisfies the following property: the sum of its edges to other vertices in the same partition is greater than the sum of its edges to other vertices in the other partition. Third, every unique gene *g* in the interaction data generates a bipartite subgraph (called a BPM) from this set of *M* bipartitions. Namely, one partition of the BPM includes *g* and every gene from the *M* bipartitions that is in the same partition as *g* at least *C*% of the time. The other partition of the BPM includes every gene from the *M* bipartitions that is in the opposing partition as *g* at least *C*% of the time. Finally, the set of all BPMs from all the genes is pruned, either to remove BPMs whose partite sets are too small or too large (see parameter settings, below), or to remove substantially overlapping BPMs generated from different genes *g* from the set.

Since a couple of the steps in the aforementioned algorithm involve many independent calculations, Genecentric parallelizes those computations automatically using the *multiprocessing* module included in the Python standard library. Therefore, large data sets can be used to generate BPMs in reasonable time. Genecentric was able to produce BPMs for a data set containing 1,685,210 genetic interactions in a few hours on an AMD 48 core Linux machine with default parameters. On a smaller data set (220,116 genetic interactions), Genecentric produces BPMs in under a minute on the same 48 core machine.

### Parameters

There are several parameters that can be set to customize Genecentric. Firstly, Genecentric can modify the underlying weights on the edges of the genetic interaction data. In particular, Leiserson et al. [5] left SGA weights unchanged, but squared E-MAP weights (retaining the sign) to speed convergence.

The user may also set *M*, the number of randomized bipartitions (default is 250), and *C*, the proportion of the time a gene must be on the same or opposite side as gene *g* in order to be included in *g*’s BPM (default is 0.9). (Recall that Leiserson’s algorithm generates a BPM for every gene in the input and prunes redundant BPMs.) Note that because Genecentric is a randomized algorithm, the set of BPMs produced will not be the same for every run; however, as *M* increases, the results will converge. Leiserson et al. [5] showed that with values of at least 250 and 0.9 for *M* and *C* respectively, different runs of the algorithm will produce similar results. *M* and *C* can be customized, but making *M* or *C* much smaller is not recommended since there could be too much variability between different runs of the randomized algorithm. Alternatively, increasing *M* can further decrease variability, but at a runtime performance cost.

By default, Genecentric will prune the BPM set returned to avoid repeating many overlapping, similar sets. But there are also several pruning options that are completely configurable in Genecentric. The first is the Jaccard index, *J* (default is 0.66), which specifies the similarity threshold of the resulting BPMs. Namely, every pairing of BPMs in the output is guaranteed to have a Jaccard index less than *J* (where the Jaccard index between two BPMs is defined as the size of the intersection between both BPMs divided by the size of the union of both BPMs). The final two pruning options, “minimum-size” and “maximum-size” (defaults are 3 and 25, respectively), filter the resulting set of BPMs so that no BPM has a module with fewer than “minimum-size” genes and no BPM has a module with more than “maximum-size” genes.

Recall that Genecentric outputs a tab-delimited file which contains a list of pairs of sets of genes corresponding to its putative generalized BPMs. This file can be used as input to GenecentricGO which performs GO enrichment analysis on the BPMs using FuncAssociate [17]. GenecentricGO can also be configured with several parameters. Of them, the most notable are the p-value cutoff (default 0.05) and the genespace. The p-value cutoff corresponds to the p-value cutoff in FuncAssociate: only sets of genes whose p-value is less than or equal to this cutoff will be returned in the results. The genespace is by default set to only the genes in the input genetic interaction data, but can be toggled to use all genes recorded for that species by FuncAssociate. GenecentricGO also provides an interface to use different species and namespaces with FuncAssociate.

Finally, GenecentricGO by default automatically employs the FuncAssociate multiple-testing correction by setting the “simulations” parameter to the greater of 1000 and the number of BPM modules, where 1000 is the default value set by FuncAssociate. This value is also capped at 10000 by FuncAssociate. (While in theory, we should be able to discover more enrichment by just setting the parameter to the number of modules in the input BPM file, we found that FuncAssociate’s stochastic simulations to estimate p-values were too variable when this parameter was set much below their default of 1000.)

## Results and discussion

We ran Genecentric on the same E-MAP dataset as discussed in the Leiserson et al. paper [5], and showed that it produced comparable results (though note that results will not be 100% identical, because, as discussed above and in their paper, results wobble slightly over different randomized runs of the algorithm). We set out next to show that Genecentric with default or nearly default parameters can be run “out of the box” to produce meaningful results that corrolate well with the biological literature on other datasets as well. In particular, a recent study [18] produced an E-MAP dataset of 374 genes involved in various aspects of plasma-membrane biology, including endocytosis, signaling, lipid metabolism and eisome function. We ran Genecentric on this E-MAP dataset using all default parameters except the weights were squared, as Leiserson et al. [5] recommends for E-MAP data, and *C* was lowered from.9 to.8 in order to produce more BPMs (22 instead of 6). Since default parameters were used, GO enrichment was computed by FuncAssociate with a genespace consisting of only genes from the E-MAP data set. Of these 22 BPMs, 7 exhibited GO enrichment in both gene sets, according to the Genecentric FuncAssociate GO module, whereas an additional 8 exibited GO enrichment in one gene set of the BPM. These enrichment rates, while good, are still somewhat below the percentage enrichment reported for the ChromBio E-MAP dataset in [5], perhaps because the component genes in this plasma-membrane E-MAP data set are not all as well studied, and thus fewer functional annotations are yet known.

**Figure 2:**
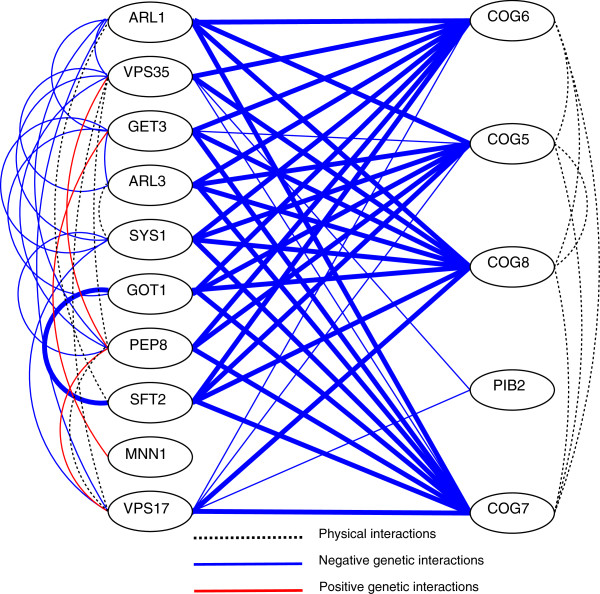
**Sample of physical and genetic interactions of a BPM.** Genetic interactions appear as solid lines. Thicker lines correspond to more negative weights. Dashed lines correspond to known protein-protein interactions. (Protein-protein interactions were taken from BioGRID’s data, with the “low-throughput” filter active).

Of the 7 BPMs that exhibit enrichment in both gene sets, 6 have one set that contains the genes coding for proteins COG 5, 6, 7, and 8, and there is some additional overlap in some of the genes in these BPMs (recall that Genecentric with default pruning options will permit overlapping BPMs provided their Jaccard index is less than 0.66). Table 1 shows a list of these BPMs and corresponding GO enrichment terms. Figure 2 depicts one of these BPMs by showing edges for physical and genetic interactions between each pair of genes.

**Table 1:** BPMs where 1 module includes the COG complex

**BPM**	**Selected enriched GO terms**
**COG6 COG5 COG8** PIB2 **COG7**	intra-Golgi vesicle-mediatedtransport (4/5)
	protein targeting to vacuole (4/5)
**ARL1 VPS35 GET3 ARL3**	protein transport (9/10)
**SYS1 GOT1 PEP8 SFT2**	Golgi apparatus (7/10)
**MNN1 VPS17**	endosome transport (6/10)
	vesicle-mediated transport (9/10)
**COG6 RIC1** MRP8 **COG5 COG7 SNC1 COG8 GCS1 PIB2 SRO7**	intra-Golgi vesicle-mediatedtransport (4/10)Golgi vesicle transport (7/10)
	vesicle-mediated transport (9/10)
**ARL1 VPS35 GET3 ARL3 SYS1 SRO77 PEP8 SFT2 GOT1 SNC2 VPS13 VPS17**	post-Golgi vesicle-mediatedtransport (7/12)Golgi vesicle transport (8/12)
	establishment of proteinlocalization (10/12)
	retromer complex (3/12)
**COG6 YPT7 COG7 MVB12 YPT52 COG5 CCZ1 ARF3 COG8 ENT3 YPT53**	intra-Golgi vesicle-mediatedtransport (4/11)establishment of proteinlocalization to vacuole (8/11)
	vacuolar transport (10/11)
	GTP binding (4/11)
**ARL1 VPS35 GET3 ARL3**	endosome transport (7/12)
**YPT31 BOR1 SYS1 GOT1**	Golgi vesicle transport (7/12)
**PEP8 SFT2 VPS17** YOL019W	retrograde transport, endosome to Golgi (4/12)
	establishment of proteinlocalization (11/12)
	retromer complex (3/12)
**COG6 COG5 COG8 COG7**	intra-Golgi vesicle-mediatedtransport (4/4)
	protein targeting to vacuole (4/4)
	establishment of prtoeinlocalization (4/4)
ARL1 **VPS35** GSF2 GET3 ARE2	retromer complex (3/10)
SFT2 GOT1 SCS7 **PEP8 VPS17**	
**COG6** RIC1 MRP8 EIS1 **ARF1 COG5** PIB2 **COG7 COG8 GCS1**	intra-Golgi vesicle mediatedtransport (5/10)
	Golgi vesicle transport (6/10)
**ARL1 VPS35** GSF2 **ARL3 SYS1 APL5 GET3 PEP8 SFT2 GOT1** ARE1 SCS7 **VPS13 VPS17**	protein transport (11/14)Golgi apparatus (8/14)endosome transport (7/14)Golgi vesicle transport (7/14)
	establishment of proteinlocalization (11/14)
**COG6 RIC1** MRP8 **VPS41** EIS1**ARF1 ARF3 COG5 YPT52 COG7 COG8 GCS1 SNC1 PIB2 ENT3**	intra-Golgi vesicle-mediatedtransport (5/15)Golgi vesicle transport (8/15)vesicle-mediated transport (13/15)
	establishment of proteinlocalization (11/15)
**ARL1 VPS35** GSF2 **ARL3**	endosome transport (9/17)
**YPT31 SYS1 VPS17 GET3**	Golgi vesicle transport (8/17)
**PEP8 SFT2 GOT1 SNC2** SCS7**VPS13** MNN1 **VPS29** ARE2	retrograde transport, endosome to Golgi (5/17)
	establishment of proteinlocalization (12/17)
	retromer complex (3/17)

This table lists several BPMs generated where one module contains genes in the COG complex. Selected enriched GO terms are included, and bolded genes have been enriched for at least one of the GO terms listed. For complete GO enrichment results, please see the Additional file 1.

 The proteins encoded by COG 5, 6, 7 and 8 constitute a structural component known of the conserved oligomeric Golgi (COG) complex, an important peripheral Golgi apparatus protein structure that has been most significantly implicated in retrograde trafficking [19] and has additional roles in supporting Golgi apparatus structure [20] and glycosylation [21]. It is a hetero-octamer consisting of two lobes, A and B, that are both comprised of four proteins, COG 1-4 for lobe A and COG 5-8 for lobe B. Recent studies have also identified COG 1 as the linking unit between the two lobes of the complex [22]. Four out of the eight COG proteins have significant homology with other organisms, including mammals, and studies have shown that mutation of COG proteins in humans can lead to a group of serious conditions known as congenital disorders of glycosylation [21,23]. Deletions in COG 1-4 (Lobe A) in Saccharomyces cerevisiae cause severe growth defects, but COG 5-8 have been shown to be non-essential [22]. Additionally, the mutations that cause these severe growth defects are phenotypically distinct, indicating different roles for the different subunits of the COG complex [24,25]. Other genes that occur multiple times in the same set as the COG proteins in the BPMs include PIB2, a protein that binds phosphatidylinositol 3-phosphate, involved in vesicle-mediated transport. Phosphatidylinositol-3-phosphate, together with small GTPases, is also an important factor for sorting in the endocytic pathway [26]. Other proteins were CCZ1, a protein involved in vaculolar transport and vesicle docking, ARF1, ARF3 and GCS1, where ARFs are GTPases of the Ras superfamily that regulate the formation of coated vesicles in intracellular trafficking [27,28] are regulated by the GTPase activating protein GCS1 [29]. RIC1, a GTPase invovled in localization of trans-Golgi membrane proteins [30] and MRP8, whose function is not known, also appear. 

On the opposite module of the BPMs containing COG proteins, frequently occuring genes include VPS35 and PEP8, components of the retromer complex needed for retrograde transport, as well as VPS17, a protein associated with proper vesicle formation. Additionally, the protein GOT1, which plays a role in secretory transport [31] was found, which further hints at the variable function of the COG complex and its subunits. The second module also contained the genes encoding for the ARL1, SYS1, and ARL3 proteins—which are involved in vesicle tethering at the Golgi apparatus [32]. It is interesting that, different from the datasets discussed in the Leiserson *et al* paper [5], a large percentage of the enriched BPMs contain the same COG complex. We don’t know if the popularity of the COG complex in our results is simply because of what annotation is known, something about the interaction of the algorithm with the distribution of the E-MAP weights, or multiple roles for the COG complex that make it especially able to compensate for multiple different biological processes.

In addition to the BPMs containing the COG complex, there are two dually enriched and seven singly enriched BPMs (see Tables 2 and 3). We note that some of the BPM modules—that do not show up as GO enriched—support functional coherence when we dig more deeply into the literature. For example, a module in one of our BPMs, RTG3 ALP1 PEP12 XRN1 BCH2 RTG2 SIW14, is not flagged as GO-enriched by FuncAssociate, but we found that RTG3 and RTG2 are known to be involved in the retrograde signalling pathway. PEP12 is a multifunctional yeast syntaxin that controls entry of biosynthetic, endocytic and retrograde traffic into the prevacuolar compartment [33]. BCH2 is a member of a complex that mediates the export of specific cargo proteins from the Golgi to the plama membrane [34].

**Table 2:** Dually enriched BPMs

**BPM**	**Selected enriched GO terms**
**SLT2 BCK1** CLC1	endoplasmic reticulum unfoldedprotein response (2/3)
**PEX1 PEX6** EDE1 SKN7 ERG4ADH1 **PEX15** ARC18 ECM33	protein import into peroxisomematrix (3/9)
	receptor recycling (3/9)
**COG6 YPT7 MVB12 YPT52 COG5 CCZ1 ARF3 YPT53 ENT3**	vacuolar transport (8/9)protein targeting to vacuole (6/9)establishment of proteinlocalization (9/9)
	GTP binding (4/9)
**VPS35 YPT31** BOR1 YOL019W **PEP8**	retrograde transport, endosome to Golgi (3/5)

This table lists BPMs which have both modules enriched and do not include all of the COG 5, 6, 7 and 8 genes. Selected enriched GO terms are included, and bolded genes have been enriched for at least one of the GO terms listed. For complete GO enrichment results, please see the Additional file 1.

**Table 3:** Singly enriched BPMs

**BPM module**	**Selected enriched GO terms**
**VPS35 GET3 ARL3 SYS1**	protein transport (7/8)
**GOT1 PEP8 SFT2** ARE1	endosome transport (5/8)
**PEX6** BZZ1 SKN7 BLS1 **PEX1**TWF1 **PEX15** ARC18 JSN1ECM33	protein import into peroxisomematrix,receptor recylcing (3/10)
**SNC1 GCS1 SRO7**	Golgi to plasma membranetransport (3/3)
**YPT7 MVB12 ARF3 CCZ1 YPT52 VPS41** STD1 **ENT3**	establishment of proteinlocalization (7/8)
**VPS27** YOL019W **VPS4**	protein retention in Golgi apparatus (2/3)
	maintenance of protein location in cell (2/3)
**RVS161 RVS167** SKN1	lipid tube assembly (2/3)
**RVS161** PKH3 **ASG7**	conjugation with cellularfusion (2/3)

This table lists enriched modules from BPMs which have only one module enriched for GO terms. Selected enriched GO terms are included, and bolded genes have been enriched for at least one of the GO terms listed. For complete GO enrichment results, please see the Additional file 1.

 A full list of Genecentric BPMs is provided in the Additional file 1.

## Conclusions

We have introduced Genecentric, a package that can automatically accept a set of high-throughput genetic interaction data, and output generalized BPMs along with their enrichment values. Genecentric has several features that make using it both easy and fast: 

• Its only dependency is Python—no compilation steps or third party libraries are required. Any system capable of running Python should also be able to run Genecentric.

• All of the parameters of the algorithm are easily configurable using Genecentric’s command line interface. (Namely, *M*, *C*, and *J* as used in the aforementioned algorithm description.) The parameters are by default set to those used in [5]: *M*=250, *C*=0.9 and *J*=0.66.

• Genecentric automatically takes advantage of multiple CPUs.

• Input data can be read directly from genetic interaction data files. The user may specify an additional set of genes to *exclude* from BPM generation (i.e., a list of essential genes).

• Genecentric is species and gene identifier agnostic. (i.e., Genecentric does not care which kind of gene identifiers are used.)

• Code is well documented and could be extended easily.

Finally, we provided an extension to Genecentric that can perform Gene Ontology (GO) enrichment on a set of BPMs. Genecentric uses FuncAssociate’s web API to achieve this, and parameters like genespace, namespace and p-value are configurable on the command line.

## Availability and requirements

**Project name:** Genecentric

**Project home page: **http://bcb.cs.tufts.edu/genecentric

**Operating system(s):** Platform independent

**Programming language:** Python

**Other requirements:** Python 2.6 or higher

**License:** GNU General Public License Version 2

**Any restrictions to use by non-academics:** No

## Competing interests

The authors declare that they have no competing interests.

## Authors’ contributions

Conceived and designed the project: AG and BH. Algorithmic development: ML, LC and BH. Implemented the software: AG. Analyzed the data: AG, MK, LC and BH. Wrote the paper: AG, LC and BH. All authors read and approved the final manuscript.

## Funding

This work was partially funded by the National Institutes of Health (grant 1R01GM080330 to LC).

## Supplementary Material

Additional file 1 **All BPMs generated with GO enrichment.** A full list of BPMs generated by Genecentric from E-MAP data with 374 genes involved in various aspects of plasma-membrane biology, including endocytosis, signaling, lipid metabolism and eisome function. Click here for file
